# Nanocellulose Based Filtration Membrane in Industrial Waste Water Treatment: A Review

**DOI:** 10.3390/ma14185398

**Published:** 2021-09-18

**Authors:** Yunxia Liu, Honghai Liu, Zhongrong Shen

**Affiliations:** 1 College of Furnishings and Industrial Design, Nanjing Forestry University, Nanjing 210037, China; xmlinyunxia@fjirsm.ac.cn; 2 Co-Innovation Center of Efficient Processing and Utilization of Forest Resources, Nanjing Forestry University, Nanjing 210037, China; 3 Xiamen Key Laboratory of Rare Earth Photoelectric Functional Materials, Xiamen Institute of Rare Earth Materials, Haixi Institutes, Chinese Academy of Sciences, Xiamen 361021, China; z-shen@fjirsm.ac.cn

**Keywords:** nanocellulose, membrane separation technology, filtration membrane, industrial wastewater treatment

## Abstract

In the field of industrial wastewater treatment, membrane separation technology, as an emerging separation technology, compared with traditional separation technology such as precipitation, adsorption, and ion exchange, has advantages in separation efficiency, low energy consumption, low cost, simple operation, and no secondary pollution. The application has been expanding in recent years, but membrane fouling and other problems have seriously restricted the development of membrane technology. Natural cellulose is one of the most abundant resources in nature. In addition, nanocellulose has characteristics of high strength and specific surface area, surface activity groups, as well as being pollution-free and renewable, giving it a very wide development prospect in many fields, including membrane separation technology. This paper reviews the current status of nanocellulose filtration membrane, combs the widespread types of nanocellulose and its derivatives, and summarizes the current application of cellulose in membrane separation. In addition, for the purpose of nanocellulose filtration membrane in wastewater treatment, nanocellulose membranes are divided into two categories according to the role in filtration membrane: the application of nanocellulose as membrane matrix material and as a modified additive in composite membrane in wastewater treatment. Finally, the advantages and disadvantages of inorganic ceramic filtrations and nanocellulose filtrations are compared, and the application trend of nanocellulose in the filtration membrane direction is summarized and discussed.

## 1. Introduction

Industrialization, growing population, and rapid urbanization have led to serious water and land pollution [1]. Wastewater mainly contains pollutants such as saturated salts, heavy metals, organic compounds, oil emulsions, dyes, and even microorganisms. Nanocellulose materials have broad prospects in wastewater purification and mitigation [2,3,4]. At present, the more effective and widely used methods for industrial wastewater treatment are various low-cost adsorbents [5]. In the field of industrial wastewater treatment, membrane separation technology is an emerging separation technology. Compared with traditional separation technologies of precipitation, adsorption, and ion exchange, membrane separation technology has many advantages [6] such as high separation efficiency, low energy consumption, low cost, simple operation, and no secondary pollution; therefore this technology has broad application prospects in removal of radioactive elements and heavy metal ions from industrial wastewater [7,8], and the extraction of rare earth elements from ion-type rare earth smelting wastewater [9].

Plants such as wood are the most abundant renewable materials in nature. For their applications, they are mainly concentrated on two aspects: reprocessing them in macrography, or decompose them into micro materials for research (such as nano materials).For example, wood is a very environmental protection and practical material, which has been widely used in construction, furniture and other fields since ancient times [10,11]. In view of how to improve the quality of wood, many scholars have made good achievements in different performance research, such as flame-retardant treatment [12], improve physical performance [13], superhydrophobic treatment [14], research on transparent wood [15,16], etc. At the micro level, wood is mainly composed of cellulose, hemicellulose and lignin, in which cellulose is the main component. 

Because of its non-toxic, renewable, and degradable properties, cellulose is currently widely used in the development and application of emerging materials. Cellulose can be converted into nanocellulose (CNs) through various physical and chemical methods. Owing to its high specific surface area and nano-size [17,18], NCs are more suitable for effective removal of pollutants than micro-size materials. The surface of NC contains a large number of free hydroxyl groups, which are easily modified by some functional groups [19]. The abundant free hydroxyl groups on surfaces of CNs are easy to form a large number of hydrogen bonds between molecules, which lead to good film-forming properties [20]. Cellulose has high crystallinity, specific surface area and mechanical strength, and large number of hydrophilic groups; thus nanocellulose is often used as a filter membrane reinforcement material to improve the mechanical and hydrophilic properties of functional membranes [21]. Meanwhile, as the most abundant raw material in nature, cellulose has lots of advantages such as broad resources of raw material, easy processing, and low cost, which lead to it having extremely broad prospects in membrane separation technology. This article reviews the application of nanocellulose in filter membranes and the research in industrial wastewater treatment using nanocellulose filter membranes, summarizes the advantages and disadvantages of the application of nanocellulose filter membranes, and finally puts forward a prospect for further research.

## 2. Nanocellulose Filtration Membrane

### 2.1. Nanocellulose (NC)

Cellulose, a renewable natural polymer compound, is a single water-insoluble polysaccharide composed of 1.4-β-D-pyran-type dehydrated polydextrose [22]. Cellulose is currently widely used in the development and application of emerging materials due to it being nontoxic, renewable, and degradable. Cellulose can be chemically and physically treated into nanocellulose (NC), which is cellulose crystals or fibers in nanoscale [23]. Nanocellulose can be divided into four categories: cellulose nanofiber (CNF), cellulose nanocrystal (CNC), electrospun nanocellulose (ECC), and bacterial synthesis of nanocellulose (BNC). Although the sources of CNF, CNC, and ECC are all plant, there are also certain differences in structure and function due to different preparation methods.

**Figure 1:**
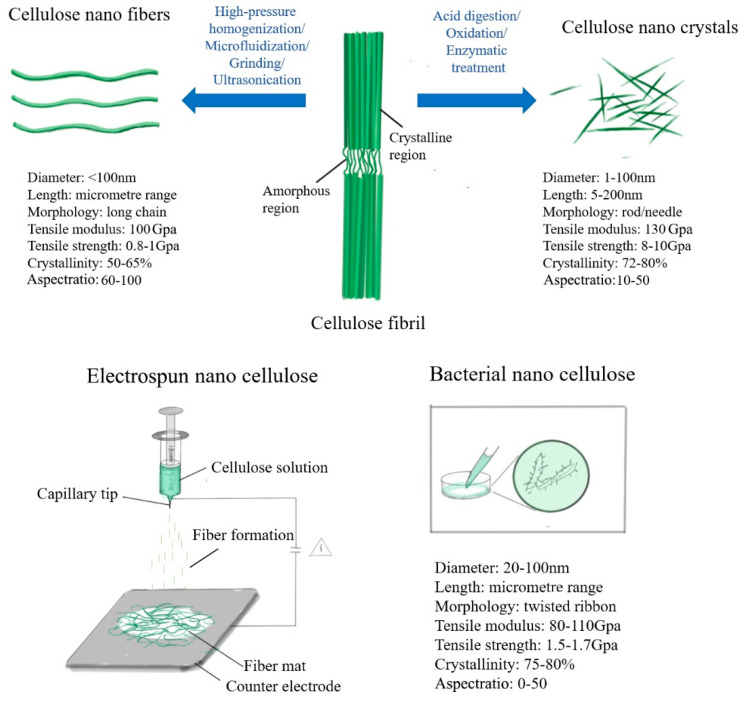
Preparation of CNF, CNC, ECC, and BNC.

CNF is obtained by separating microfibrils (bundles) in animal and plant fibers through mechanical shearing or chemical oxidation. The more mature process is chemical-mechanical combined treatment [24]. CNC is a nanometer-sized cellulose crystal produced by hydrolysis of cellulose, and an aqueous CNC suspension is usually produced by acid hydrolysis (Figure 1). Ranby et al. [25] first reported the process of preparing CNC by acid hydrolysis in 1951. The method was then further optimized. The main principle is that acid hydrolysis destroys the amorphous regions of cellulose, while retaining the crystalline regions with higher crystallinity [26]. Filtration, centrifugation, or ultracentrifugation is needed to obtain a uniformly dispersed CNF aqueous solution [27,28]. Nanocellulose of ECC and BNC are classified via preparation and prevention. ECC is a kind of nanocellulose prepared by the electrospinning method. Nanofibers prepared by electrospinning technology have large specific surface area, high mechanical strength, and broad application prospects in medical and pharmaceutical fields [29]. ECC preparation can be roughly divided into two ways: one is firstly using electrospinning to prepare cellulose derivatives, and then hydrolyzing the cellulose derivatives to prepare ECC [30,31]; another is dissolving cellulose in a suitable solvent, then using electrospinning technology to prepare ECC [32]. The biggest difference between BNC and the other three types of nanocellulose is its source. CNF, CNC, and ECC are all derived from plants, while BNC is derived from microorganisms. BNC is a kind of nanofiber synthesized by aerobic bacteria [33]. Currently, the main strain synthesis of BNC is acetobacter xylinum. The preparation methods of CNF, CNC, ECC, and BNC are shown in Figure 1.

**Figure 2:**
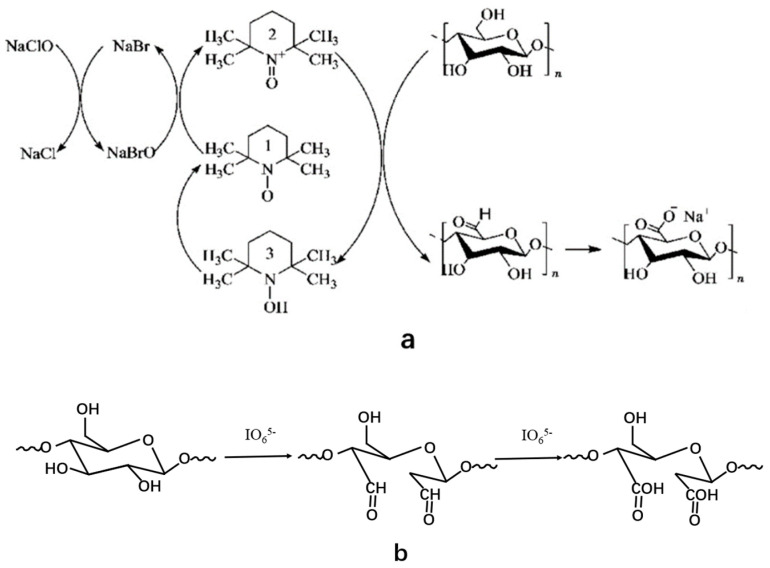
(**a**) TEMPO system oxidizes cellulose mechanism; (**b**) periodate oxidation mechanism.

The principal methods of chemical modification of nanocellulose include oxidation, esterification, etherification, cross-linking, and graft copolymerization. Cellulose oxidation is divided into selective oxidation and non-selective oxidation. Non-selective oxidants include sodium hypochlorite, persulfuric acid, and hydrogen peroxide. These oxidants can cause the oxidation of hydroxyl groups on cellulose, and the oxidative degradation is severe and difficult to control; selective oxidation is the oxidation of -OH in a specific position of cellulose, thus it can effectively inhibit the degradation of cellulose during oxidation [34]. The 2,2,6,6,-tetramethylpiperidine oxide oxidation system (TEMPO/NaBr/NaClO) and periodate have been widely studied in the field of oxidized cellulose materials because of their high efficiency, economy, and environmental protection [35]. The TEMPO/NaBr/NaClO system only selectively oxidizes the primary hydroxyl groups of cellulose but has no effect on the secondary hydroxyl groups and it can be recycled and regenerated with a simple reaction process and high selectivity. It is usually carried out in the condition of alkaline medium [36]. The mechanism of oxidizing C6 primary hydroxyl of cellulose using TEMPO/NaBr/NaClO system is shown in Figure 2 [37]. Unlike the TEMPO system, periodate only oxidizes the secondary hydroxyl groups of cellulose, breaking the chemical bond between C2 and C3, then forming two aldehyde groups. Sodium periodate (NaIO_4_) and potassium periodate (KIO4) are considered to be the most effective oxidants for the selective oxidation of cellulose C2 and C3 hydroxyl groups. The aldehyde content of oxidized cellulose can reach more than 97% [38,39].

Esterified cellulose is formed by a series of condensation reactions between -OH on cellulose and acid, acid anhydride and acid halide, etc. The traditional industrial production of cellulose esters basically adopts a solid–liquid two-phase two-step process for lack of effective cellulose solvents: firstly, fully substituted cellulose esters are obtained through solid–liquid two-phase heterogeneous acylation, and then cellulose esters with appropriate degree of substitution is obtained by acid catalytic hydrolysis [40,41].

Etherified cellulose refers to a series of derivatives formed by the reaction of -OH on cellulose chain with alkylating reagents in an alkaline medium. Since the cellulose etherification reaction must use a strong base as a catalyst, the LiOH/urea and NaOH/urea aqueous solution systems are very suitable homogeneous etherification reaction media. The reaction conditions are mild, fast, and efficient, and do not require catalysts and other organic solvents [42,43]. 

**Figure 3:**
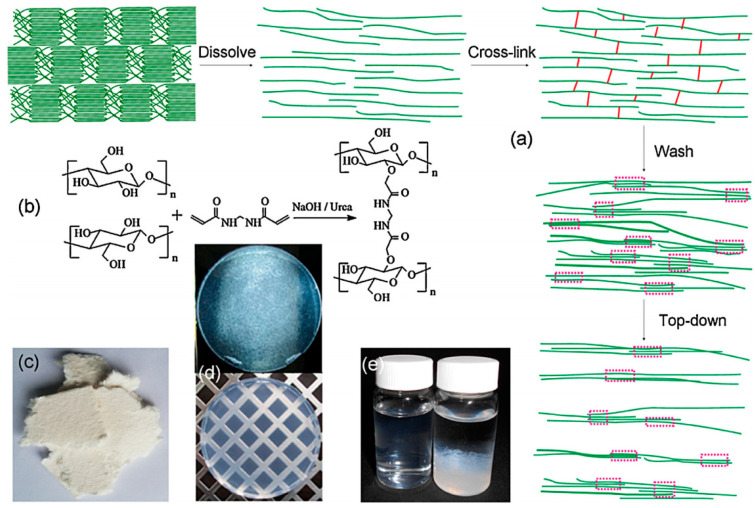
(**a**) Schematic diagram of hydrogel prepared by dissolving and cross-linking cellulose; (**b**) chemical equation of cellulose cross-linking reaction in NaOH/urea aqueous medium; (**c**) photo of hydrogel after washing of cross-linked cellulose; (**d**) the image on the left is an aqueous dispersion of chopped cellulose hydrogel, and (**e**) the image on the right is a distilled water dilution chart of the cellulose solution [37].

Cross-linked cellulose refers to the product with a three-dimensional network structure by cross-linking points between cellulose, cellulose derivatives, or other high polymers using cross-linking agents such as epichlorohydrin (ECH), N, N’-methylene bisacrylamide (MBA), etc. As shown in Figure 3, cellulose gel can be produced through MBA cross-linking, and the hydrogel has high transparency (Figure 3c). Compared with the direct water dispersion of cellulose solution, the cellulose hydrogel prepared by ultrasonic cutting after cellulose cross-linking has good dispersibility in water, the dispersion solution is transparent, and there is no flocculation [44,45] (Figure 3d). Graft copolymerization is an important method for cellulose chemical modification, which can give cellulose new characteristics. According to the type of polymerization reaction, cellulose graft copolymerization could be divided into radical polymerization, ion polymerization, ring-opening polymerization (ROP), atom transfer radical polymerization (ATRP), reversible addition fragmentation chain transfer polymerization (RAFT), nitrogen and oxygen stabilized free radical polymerization (NMP), etc. [46,47].

### 2.2. Membrane Separation Technology

Membrane separation technology refers to a technology that can achieve selective separation when a mixture of particles with various diameters at the molecular level passes through a filter membrane. The core component of membrane separation technology is a natural or synthetic filter membrane, which has good selective permeability, and can separate, purify, and enrich two-component or multi-component solutes and solvents through external energy or chemical potential difference as the driving force. At present, filter membranes can be divided into five categories according to driving pressure (Table 1) which are microfiltration (MF) [3], ultrafiltration (UF) [48], nanofiltration (NF) [49], reverse osmosis (RO) [50], and forward osmosis (FO) [51]. Compared with the forward osmosis where the driving force is the penetration pressure difference on both sides of the solution, the reverse osmosis technique is that the solvent overcomes the pressure difference driven by an external force.

A cellulosic membrane is a kind of membrane material which was studied and applied earliest and most widely used currently. Nitrocellulose (CN) is made by nitrification of cellulose and is widely used in dialysis membranes and microfiltration membranes. CN can also be mixed with cellulose acetate to increase its strength [56]. Cellulose diacetate (CA) and cellulose triacetate (CTA) are the basic materials for the preparation of RO membranes, and they are also used in the application fields of UF, NF, and MF. Ethyl cellulose (EC) is produced by the reaction of alkali cellulose and ethyl halide, and is often used for nitrogen and oxygen separation. In addition, other cellulose-derived materials such as cellulose acetic acid and mixed esters of butyric acid are also used in membrane materials.

Membrane separation as the core technology has gradually been widely used in the field of water treatment. The “ultrafiltration + nanofiltration + concentrated water reverse osmosis” membrane treatment with nanofiltration membrane as the key technology has been widely and steadily applied in the field of drinking water purification fields such as water plants. Seawater contains inorganic salt ions (such as calcium ions) that are difficult to remove. The study on seawater desalination technology is of great significance to seawater utilization. In current seawater desalination, reverse osmosis technology can be effective to remove carbonate and bicarbonate from seawater when adding inhibitors and acids at the same time [57]. In addition to the application in drinking water, membrane separation technology also has greater prospects in industrial wastewater treatment. For example, the removal of radioactive elements [7], the removal of heavy metal ions, and the extraction of rare earth elements from ionic rare earth smelting wastewater [9]. In the field of water treatment, membrane separation has become one of the core technologies due to its unique advantages. However, the pollution of the membrane and other problems have severely restricted its further development [58,59]. How to solve the pollution of the membrane is also a direction of further research for scholars.

### 2.3. Nanocellulose Filtration Membrane

Because cellulose has high crystallinity, high specific surface area and mechanical strength, and a large number of hydrophilic groups on the surface, nanocellulose is often used as a filter membrane reinforced composite material to improve the physical properties of functional membranes such as mechanical strength, hydrophilicity, etc. [60]. Mokhena et al. [61] prepared a nanofiber composite membrane by coating the extracted corn stalk nanowhiskers (CNs) on electrospun alginate nanofibers treated with CaCl_2_. They found that the membrane can completely remove water pollutants of 10–100 nm particles, and the retention rate of chromium (Cr(VI)) is 80% at pH 11. This indicates that the membrane can be used for short-term wastewater treatment and/or domestic water purification. Ma et al. [62] functionally improve traditional commercial filter membranes using TEMPO oxidized nanocellulose and prepared microfiltration and nanofiltration membranes with a 2–10 times higher membrane flux than that of commercially available membranes.

In addition, nanocellulose and its derivatives are also used as the matrix material of the filter membrane due to its special advantages such as being, green, pollution-free, and recyclable, as well as good film-forming properties. Hugo et al. [4] studied the filtering performance of CNF membranes for particles with various diameters. Furthermore, in view of the deficiencies of the cellulose film, such as small membrane flux, low retention rate, and low service life, some scholars have tried adding nano-unit silica particles to modify it. Varanasi [63] prepared a cellulose nanofiber composite membrane through filtering the suspension of cellulose nanofibers, SiO_2_ nanoparticles (22 nm), and polyamide amine-epichlorohydrin (PAE). The silica nanoparticles were used as a spacer to control of the pore size of the nanofiber network, which controlled the pore size of the membrane and thus improved the flux of the membrane, while PAE can make the negatively charged nanoparticles adhere to the nanofibers and improve the wet strength of the membrane.

A large number of free hydroxyl groups on the surface of nanocellulose can easily react with other chemical reagents, thus improving its various properties. Nanocellulose are often used as a carrier or a framework material in the filter membrane, which are combined with other materials to form a composite filter membrane. Ma [64] and other research groups have invented a new type of TFC membrane, which contains various fiber structures with various diameters and lengths. This new structure is called a thin-layer nanofiber composite (TFNC) structure. The nanofiber composite membrane is usually composed of three layers: the bottom layer is a conventional nonwoven substrate (polyethylene terephthalate, PET non-woven fabric mat); the middle layer is an electrospun nanofiber scaffold which replaces the porous layer prepared by the traditional phase transformation method; the top filter layer could be a hydrophilic polymer coating or another finer nanofiber layer. The TFNC membrane combines the property of high porosity of electrospun nanofibers and hydrophilic filter layer, which greatly improves the permeate flux, and is widely used in the field of UF [65,66]. The TFNC membrane with nanocellulose filter layer maintains the same retention rate, while the permeate flux is 5–10 times that of commercial ultrafiltration membranes [67,68,69,70].

## 3. Application of Cellulose Filter Membrane in Industrial Wastewater Treatment

The main sources of industrial wastewater pollutants are heavy metals, suspended solids, polycyclic aromatic hydrocarbons, and biomolecular pollutants [71]. NC has great advantages in wastewater treatment due to its excellent specific surface area and mechanical strength, adjustable surface chemistry, surface groups for cationic or anion selective grafting, and hydrophilicity [72].

Functionalized CNF could be used to extract oil and organic pollutants from wastewater [73]. Membrane fouling occurs on the surfaces. Generally, the biofilms are formed on the surface of the membranes due to non-specific interactions between the membrane surface and pollutants. The membrane performance of permeability and selectivity are reduced because of the biofilm formation or clogging of membrane pores. The CNC-based nanocomposite membrane has good hydrophilicity, porosity, and surface permeability, which makes it have good antifouling performance. The surface modification of NC can improve the adsorption performance for various pollutants in aqueous solution and guide its selectivity. The possible reason is that the available active binding sites are increased after modification, thereby improving the ion exchange characteristics and generating new functional groups that promote the absorption for the metal backbone [1,74]. Table 2 shows the application of some nanocellulose and their derivatives in wastewater treatment.

At the same time, how to increase the membrane flux as much as possible while ensuring the rejection rate is still a problem that many scholars are trying to solve. At present, in order to improve the permeability of the filtration membrane, in addition to adding a hydrophilic modifier, increasing the specific surface area of the filtration membrane is a very effective method for improving the permeability [75,76]. Teng prepared CNF-supported high-water content polyamide (PA) nanofiltration membranes with an arched structure through the interfacial polymerization (IP) method. During the IP process, the hydration of BCN promoted Marangoni along the water/organic solvent interface. Convection and produce a thin PA active layer with an arch bridge structure. These arch bridge structures enable the resulting PA active layer to have a significantly larger active area to achieve water penetration. Therefore, the PA NF membrane exhibits excellent desalination performance, with a permeability as high as 42.5 Lm^−2^ h^−1^ bar ^−1^ and can remove Na_2_SO_4_. The permeability is as high as 99.1%. The total desalination performance is better than almost all reported so far for existing NF membranes.

### 3.1. Research on NC as a Membrane Matrix Material in Industrial Wastewater Treatment

The application of NC as a matrix material in filter membranes mainly includes two aspects: 1. NC filter membrane; 2. NC polymer composite filter membrane [90]. There are three main ways to prepare NC membranes: impregnate electrospinning scaffolds with NC; impregnate coating after vacuum filtration of NC; self-assembly films forming after NC solution losing water.

Phase inversion technology is an efficient method for the preparation of asymmetric membranes. Lingling [91] prepared cellulose triacetate (CTA) ultrafiltration membranes by phase inversion, and then used TEMPO oxidized cellulose (TOCNs) as hydrophilic modification. The filtration membrane was modified and characterized separately, and the effect of TOCN on the performance of the CTA filtration membrane was analyzed. The results showed that the addition of TOCN greatly improved the properties of hydrophilic and mechanical of the filtration membrane, which improves the antifouling performance of the membrane.

CNC composite film has a high density of negative surface charges, resulting a strong adsorption capacity for positively charged dyes. Studies have shown that, compared with commercial MF membranes, NC doped with MF membranes has higher dye adsorption efficiency [92,93]. Ma et al. [81] also coated nanocellulose (NC) on the surface of PAN electrospun nanofiber membranes. The composite nanofiber membranes can be used to remove aquatic viruses, such as MS2 (bacteriophages), showing a high retention rate. Meanwhile, due to the carboxyl, hydroxyl, aldehyde, and other functional groups on the surface of cellulose and its derivatives, nanocellulose can form a new nano-network structure in electrospun nanofibers, this resulting high adsorption performance, and retention rate of the composite nanofiber membranes. Compared with traditional membranes, the composite membrane structure has abundant functional groups on the surface of nanocellulose as adsorption sites to remove contaminants such as viruses, dyes, heavy metal ions, and toxins, providing more applications of the ultrafiltration membrane in industrial wastewater treatment. Compared with commercial membranes (PAN10 and PAN400) at the same working pressure, the nanofiber composite membrane maintains high permeation flux and high retention rate. Goetz et al. [94] prepared CA membranes by electrospinning, and then impregnated the CA membranes in different concentrations of CNC solutions. The membranes showed a nanostructured surface post impregnation, and the mechanical properties were significantly improved. The contact angle of nanofiber composite membranes was reduced to 0° from 102° of the original CA membranes. This indicates that nanofiber composite membranes have completely hydrophilic property. In addition, the membranes also exhibit a high adsorption capacity of 80–99% for dyes.

The surface of CNF contains a large number of hydroxyl groups, which are interwoven into a colloidal form in the aqueous solution. During the water losing process of CNF solution, the hydroxyl groups between adjacent nanofibers combine to hydrogen bonds and then form a dense membrane, which is a CNF self-assembled membrane. The membrane has excellent mechanical properties, and its permeability is far lower than that of high and low-density polyethylene membrane with the same thickness [95]. Hassan et al. [96] subsequently reported the papermaking wastewater treatment using a filter membrane made by copper terpyridine modified oxidized cellulose nanofibers (OXCNF-Cu-Tpy). The OXCNF-Cu-Tpy was prepared by modifying the OXCNF using 4′-chloro [2,2′,6′,2″] terpyridine copper(II). The modification was verified by elemental analysis and Fourier transform infrared spectroscopy. The results showed that the pure water flux of OXCNF-Cu-Tpy was 30% higher than that of the unmodified OXCNF membrane. Mokhena et al. [61] prepared a nanofiber composite membrane by coating the extracted corn stalk nanowhiskers (CNs) on electrospun alginate nanofibers treated with CaCl_2_. They found that the membrane can completely remove water pollutants of 10–100 nm particles, and the retention rate of chromium (Cr(VI)) at pH 11 is 80%, which indicates that the membrane can be used for short-term wastewater treatment and domestic water purification.

Cellulose acetate is one of the most widely used ultrafiltration membrane materials for industry [97]. The adsorption and deposition of biological macromolecules on surfaces and inside pores of the membrane cause serious pollution, this greatly limits the efficiency and application of ultrafiltration [58,59,98]. Therefore, almost all research on ultrafiltration focuses on improving its antifouling performance and pure water flux, especially introducing hydrophilic materials into ultrafiltration membranes, while cellulose and its derivatives are ideal hydrophilic modification materials due to the rich hydrophilic groups on the surface. Presently, there have been many studies on the modification and enhancement of cellulose acetate filter membranes based on CNF and CNC [99,100]. Battilola [101] prepared asymmetric membranes based on CA and CNF through a phase inversion method, and studied the effect of CNF addition on the shape of CA membranes, water flux, and filtration performance. The results showed that the pore size and pure water flux of the filter membrane increased with the increase of CNF. Meanwhile, its filtration performance is fully satisfied for the clarification of juice whey. Zhou et al. [102] used CNC as a modified additive to prepare blended membranes using CNF and CA blend phase inversion method, and measured the porosity, hydrophilicity, pure water flux, tensile strength, and antifouling activity of the membrane before and after modification. The results indicated that the addition of CNC can improve the pure water flux and the porosity of the CA membrane, and can greatly improve the antifouling activity and tensile strength of the CA membrane.

### 3.2. Research on CN Composite Membranes in Industrial Wastewater Treatment

The composite membrane comprises at least two membrane structures—a porous support layer and a dense filter layer [103]. Because nanocellulose is easy to form a dense membrane structure and its excellent hydrophilicity, it is often used as a dense layer structure for preparing composite filter membranes.

A membrane with high porosity structure can be prepared using nanocellulose by some special methods and can also be used as a porous support layer for composite membranes. Yoon et al. [62] prepared thin-layer nanofiber composite (TFNC) membranes using electrospun nanofiber scaffolds as substrates. The porosity of the electrospun nanofiber is 80 to 95%, presenting high porosity characteristics; thus the electrospun nanofiber membranes are used as high-flux membranes. Wang et al. [104] composited nanofiber membrane using cross-linked polyethylene glycol (PEG) and ultrafine nanocellulose (CN), which can filter a bovine serum albumin (BSA) solution. The water flux of the membrane is approximately twice that of commercial membranes, and the retention rate remains above 90%. The regenerated cellulose membrane prepared from trimethylsilyl cellulose was studied and used to treat artificial dye wastewater [105]. The results showed that the membrane reached a flux of 600LMH at 80 °C and 4 bar and maintained a nearly 98% high dye retention rate. In the extended experiment, the membrane showed good antifouling activity up to 75 h, and the flux recovery was close to 100%. This research may provide a promising alternative method for dye wastewater treatment with a large amount of monovalent salts.

Interfacial polymerization (IP) technology is an efficient method for preparing composite filter membranes that has been widely used in recent years. Wang et al. [104] studied a high-flux nano-filtration membrane using the thin-film nanofibrous composite (TFNC) based on the interfacial polymerized polyamide barrier layer on the polyacrylonitrile (PAN) nanofiber scaffold. The results showed that under the same chemical composition, the permeation flux of TFNC membrane is 2.4 times higher than that of conventional composite membrane. Later, Teng et al. [106] used nanocellulose to improve the structure of the TFNC membrane. In a high-humidity salt solution, they prepared a polyamide (PA) nanofiltration membrane (PANF) with arched structure by the IP method based on the BNC/PTFE (polytetrafluoroethylene) composite membrane. They found that the pure water flux of the PANF membrane is as high as 42.5 Lm^−2^h^−1^bar^−1^ and the retention rate of NaSO_4_ is as high as 99.1%. Its osmotic selective desalination capacity is stronger than any of the membranes currently reported. Yung et al. [107,108] have prepared TFNC membrane using cellulose nanofibers by IP method and obtained good results. Using polyethylene terephthalate (PET) as a non-woven substrate supporter, Ma et al. [81] prepared a MF membrane using reinforced composite electrospinning polyacrylonitrile (PAN) by dipping method. The absorption rate of MF membrane to cationic dye is 16 times that of the commercial MF membrane and the retention rate of bacteria and other particles is also excellent. Wang et al. [53] coated an appropriate amount of CNCs on the surface of PES microfiltration membrane as an intermediate layer and then prepared a nanofiltration membrane using interfacial polymerization method. The experiment showed that the permeability of the membrane using modified CNCs as intermediate layer has been greatly improved. The pure water penetration rate is 34 Lm^−2^h^−1^bar^−1^, and the retention rate of sodium sulfate is above 97%. 

## 4. Comparison of Inorganic Ceramic and CN Membranes

An inorganic filtration membrane is a solid separation membrane with selective permeability made of inorganic materials such as metals, ceramics, porous glass, zeolites, metal oxides, etc. Among them, the most widely applicable materials are ceramic materials. The structure of ceramic filtration membranes can be divided into symmetrical and asymmetrical structures. Compared with traditional filtration devices, ceramic microfiltration membranes with symmetrical structures have relatively small pores ranging from 0.01 to 10 μm, the separation efficiency of the ceramic filtration membrane depends on the pore size distribution of the filtration membrane and the size of the particles to be separated [109]. 

**Figure 4:**
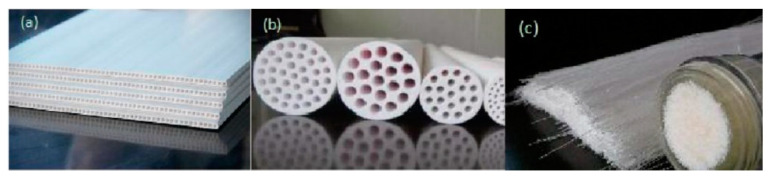
(**a**) Flat membrane; (**b**) tubular membrane; (**c**) hollow fiber membrane.

Generally speaking, the flux of the filtration membrane is inversely proportional to the thickness of the filtration membrane, that is to say, the greater the thickness of the filtration membrane, the smaller the flux of the filtration membrane. The greater the flux of the filtration membrane, the higher the separation efficiency, and the lower the time and economic cost. Therefore, the ideal filtration membrane thickness should be thinner. However, the thinner the filtration membrane is, the lower its mechanical strength is, and it is easy to be damaged during the filtration process or the cleaning process. Therefore, researchers have developed an asymmetric filtration membrane structure. It is mainly to coat a very thin filtration layer on a support with relatively high mechanical strength, which significantly increases the flux of the filtration membrane while ensuring the mechanical strength of the filtration membrane. This is also one of the important advancements made in membrane preparation technology in the past few decades [109]. The thickness of commercial ceramic microfiltration membranes is usually about 10–20 μm. According to the shape of the support, the membranes can be divided into flat, tubular, and hollow fiber shapes. The corresponding ceramic membranes are called flat membranes, tubular membranes and hollow fiber membranes. The structure is shown in Figure 4.

### 4.1. Inorganic Ceramic Filtration Membrane

Ceramic microfiltration membranes and ultrafiltration membranes are relatively widely used in the food processing industry and industrial wastewater treatment. Laboratories, pilot projects, and some business cases show that ceramic membranes have great potential in drinking water production and large-scale urban sewage treatment. However, the current production cost of ceramic filtration membranes is relatively high, and it is difficult to meet market demand. Therefore, reducing the production cost is one of the important development directions of ceramic filtration membranes [110]. The high cost of preparation of ceramic filtration membranes mainly comes from two aspects: (1) the cost of raw materials is higher; (2) the energy consumption of the preparation process is higher [111].

**Figure 5:**
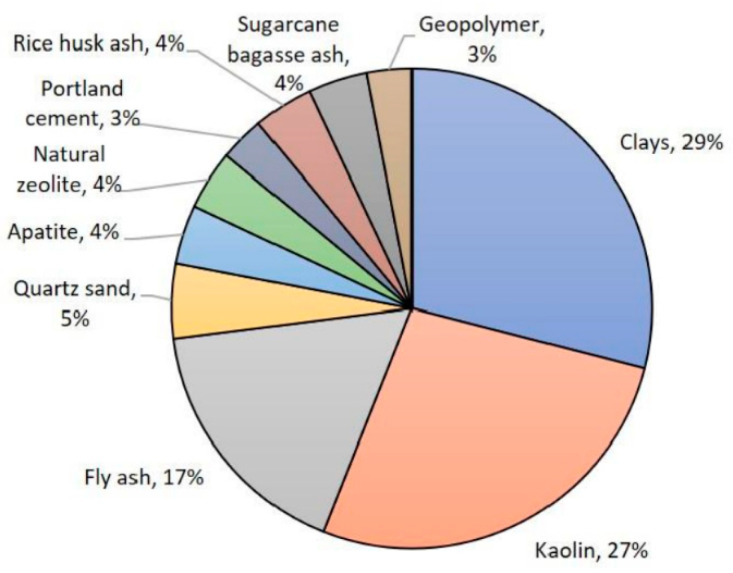
The proportion of low-cost ceramic membrane materials reported in the literature.

The raw materials of ceramic filtration membranes are mostly high-purity inorganic oxides such as Al_2_O_3_, SiO_2_, ZrO_2_, and TiO_2_, which are suitable for food and medicine filtration, while its preparation cost is relatively high. For wastewater treatment with relatively low safety-level requirements, such as oily wastewater, printing, dyeing, wastewater, etc., the necessity of high-purity oxides is questionable. For these applications, more and more researchers have used relatively cheap raw materials in recent years, including kaolin, clay, fly ash, apatite, quartz sand, mullite, natural zeolite, etc. [112] (Figure 5). For example, Bouazizi et al. [113] mixed bentonite particles (particle size <45 µm, 95 wt%) and pore former (starch, 5 wt%) uniformly, and prepared a flat support body by dry pressing. After drying and firing at 950 °C, the resulting support has a pore size of about 1.70 µm and a porosity of 32.12%. It can be used for filtration printing and dyeing and tanning wastewater. The suspended solids removal rate is 94% and 99%, respectively.

In addition, reducing energy consumption in the preparation process is also a way to reduce costs. The raw materials of ceramic filtration membranes are mostly high-purity oxides, and the firing temperature is usually higher, which increases energy consumption. In order to lower the firing temperature, sintering aids such as kaolin, potash feldspar, and titanium oxide can usually be added. Wang et al. [114] used CuO and TiO_2_ as sintering aids to reduce the firing temperature of α-Al_2_O_3_ hollow ceramic fiber membranes. When the CuO content is 3 wt% and the firing temperature is 1250 °C, the prepared ceramic membrane has a porosity of 34.6%, an average pore size of 700 nm, and a pure water flux of 1255 Lm^−2^h^−1^bar^−1^.

To improve the membrane flux of ceramic membranes, common methods are the pore former method and template method. The principle of the pore former method is to increase the porosity by adding an organic or inorganic pore former during the molding of porous ceramics, and the pore former decomposes to form pores during firing, thereby increasing the porosity. Liu et al. [115] studied the influence of different particle sizes of graphite on the porosity of SiC porous ceramics and found that when 25% of 10 μm graphite pore former was added, the porosity of SiC ceramics increased from 28% to 44%. The type of pore former also has a great influence on the porosity of porous ceramics. The main principle of the template method is to control the accumulation of ceramic particles through a regular and uniform pore former to prepare a porous material with an orderly and uniform pore structure and increase the porosity. Ahmad et al. [116] used polymethacrylate (POEM) as a template and combined dipping-lifting and sol-gel methods to prepare TiO_2_/Al_2_O_3_ composite ceramic membranes (membrane pore size of about 100 nm).

### 4.2. Comparison of Inorganic Ceramic Membranes and CN Filtration Membranes

The proper selection of wastewater filtration membranes depends on a number of factors, such as productivity, service life, cost, separation selectivity, and chemical and mechanical integrity under operating conditions [117]. Compared with cellulose-based membranes which usually work at low temperature, ceramic membranes can work at high temperature; meanwhile the ceramic and other inorganic filtration membranes have excellent pH tolerance and strong resistance to chemical degradation [118,119,120]. The disadvantages of ceramic wastewater treatment are high investment cost and high treatment temperature.

Generally, the advantage of NCs that are different from the microstructures is the large length-to-diameter ratio, high porosity, and improved internal diffusion. In addition, the NC-based porous membranes have lower density, excellent mechanical properties, and higher specific surface area and pore volume. Due to their low toxicity and carbon emissions, CNCs are a viable and renewable option that could replace most adsorbent materials for wastewater treatment [121]. Table 3 shows a detailed comparison between cellulose-based membrane filtrations and ceramic membranes.

## 5. Conclusions

NC can be easily modified on the surface to generate new binding sites and provide specific characteristics to adsorb different types of pollutants. The synthesis and modification methods of NC membranes are different, no matter if CNC, CNF, or BNC was transformed into the membrane itself or as their composite material. The advantages of NC filtration membrane are its high porosity and good hydrophilicity, high mechanical stability, excellent chemical inertness, and antifouling performance, which makes it an advantage in industrial wastewater treatment. The use of nanocellulose for modification can improve the performance of the filtration membrane in terms of separation and adsorption, especially the adsorption capacity of heavy metal ions and dyes.

This review has attempted to provide a broad vision of application of nanocellulose in filtration membranes in industrial wastewater treatment, and summarizes the advantages and disadvantages of the application of nanocellulose filtration membranes, in order to stimulate the increasing interest in the nanocellulose filtration membranes research and developments. 

In terms of performance, the filtration efficiency of nanocellulose membranes is approaching commercial membranes, but nanocellulose is often used as an important functional composite material in membrane separation technology, rather than a matrix material. The reason is due to nanofibers. Because its cost is much higher than other polymer matrix materials, due to economic considerations it cannot be the preferred matrix material. Therefore, in recent years, nanocellulose-based functional filtration membranes are usually mixed membranes containing two or more functional agents. The next phase of NC-based filtration membrane research should focus on hybrid membranes, using NC and other nanomaterials to improve adsorption capacity. In addition, more research is needed to develop nanoscale NC hybrid composite materials that can interact with different pollutants at the same time.

Therefore, the current development focus of membrane separation technology based on nanocellulose is still focused on its use as an important modification additive to coat or modify the membrane to improve membrane performance. Because nanocellulose and its derivatives have high hydrophilicity, they can improve the hydrophilicity of the filtration membrane, increase the membrane flux, and reduce membrane pollution, thereby increasing the service life and efficiency of the filtration membrane. However, the current technical problem in the nanocellulose-based membrane separation technology is still how to ensure the high membrane flux of the filtration membrane, increase the rejection rate of the filtration membrane according to different filtration conditions, reduce membrane pollution, and ensure the filtration membrane service life. In addition, how to reduce the material cost of nanocellulose to improve its economic utilization is also a corresponding difficulty that needs to be further overcome. In the future, we can focus on simplifying the production process of raw materials and optimizing the filtration membrane manufacturing technology to achieve the purpose of reducing costs.

## 6. Patents

This section is not mandatory but may be added if there are patents resulting from the work reported in this manuscript.

## Figures and Tables

**Table 1:** Classification and characteristics of the cellulose-derived membrane separation process.

Type of Filtration Membrane	Filtration Process	Pore Size (μm)	Operating Pressure (psi)	Compounds Separated	References
CNC/Polyethylene Glycol	MF	1.1–0.01	15	oil, large solids, clay	[3]
Cellulose acetate/Polysulfone	UF	0.01–0.001	50	starches, proteins, heavy metals	[52]
CNC/Polyamide50/Polyethersulfone	NF	0.001–0.0001	87	salts, mono or divalent ions	[53]
CNC/Poly(acryloyl hydrazide)	RO	<0.0001	225	heavy metals, monovalent salts	[54]
HTI cellulose triacetate	FO	0.0004–0.0001.	290	oils, desalination, heavy metal	[55]

**Table 2:** Application of nanocellulose and its derivatives in wastewater treatment.

Application	Nanocellulose		Craft	the Degree of Separation	The Type of Wastewater	References
Extraction of metals ions	Ag^+^	CNF/CNC sulfonation/CNC phosphorylation	Sorption	34.38 mg/g/	wastewater of Ag^+^	[8,77]
	Cd^2+^	CNF succination/CNC-xanthate	Sorption	0.72–1.95 mmol/g/ 154.26 mg/g	aqueous solutions of Cd(II) ions	[78,79]
	Ni^2+^	CNF-CRBOX/CNF TEMPO oxidation	Sorption	55 mg/g	waste pulp wastewater	[78,80]
	Zn	CNF-NT-MOD/CNF-CRBOX	Sorption	135 mg/g	wastewater of Zn^2+^	[78,80]
	Fe^3+^	CNF-P and CNC-P	Sorption	99% removal of and Fe^3+^	the mirror making industry	[77]
Extraction of dyes from water	CNMs based nanocomposites: CNC-PAN/CNC-maleic anhydride/CNC-hydrolyzed polyacrylamide/CNF-GTMAC/dialdehyde-CNC polyvinylamine		MF Membrane/Sorption/Sorption/Sorption/Sorption	16 times higher adsorption capacity over a commercial nitrocellulose-based MF membrane./30.0 to 348.9 mg/g/ had an adsorption efficiency of more than 90%	positively charged dye/crystal violet, methylene blue, malachite green and basic fuchsin/methylene blue (MB) dye/anionic dye/869.1 mg/g, 1469.7 mg/g, 1250.9 mg/g	[81,82,83,84,85]
	functionalized CNMs: CNC-S/CNC ammonium persulfate oxidation/CNC obtained by sulfuric acid hydrolysis/CNC-carboxylation		Sorption/Sorption	16 times higher adsorption capacity over a commercial nitrocellulose-based MF membrane/118 mg/g	positively charged dye/cationic dyes	[82,86]
Hydrocarbon (oil)/water separation	BNC tri-methylsilylation reaction with trimethyichlorosilane		Sorption	185 g/g	organic solvents and oils	[87]
Removal of virus and bacteria	two-layered PET/nanoscale PAN fibrous scaffold/ultrafine CNF		MF membrane	100 mg Cr (VI) or 260 mg Pb (II) per gram	MS2 virus	[88]
	PET/PAN fibrous scaffold/cellulose nanowisker		MF Membrane	16 times higher adsorption capacity over a commercial nitrocellulose-based MF membrane	bacteria	[81]
	cladophora algae derived CNF filtration paper		MF Membrane		xenotropic murine leukemia virus	[89]

**Table 3:** Comparison of the advantages and disadvantages of nanocellulose membranes and ceramic membranes.

Membrane Type	Advantage	Disadvantage
Nanocellulose membrane	High flexibility	Usually shorter life span
	Light in mass	Prone to membrane fouling
	Lower cost	Weak chemical resistance
	Low working temperature	Weak high temperature resistance
	Low energy consumption	
	Raw materials are environmentally friendly and nontoxic	
	Simple manufacturing method	
	Reusable and recyclable	
	Has strong mechanical properties	
	High wastewater treatment efficiency	
	Easy to operate	
Ceramic/metal membrane	High thermal stability	Inflexible
	Strong chemical resistance	High investment cost
	High pressure	Low degradability
	Long life	Less selectivity based on aperture
	Compared with the CN base, it is less likely to be contaminated	Cumbersome to make
		High energy consumption
